# A case of penicillium marneffei infection involving the main tracheal structure

**DOI:** 10.1186/1471-2334-14-242

**Published:** 2014-05-07

**Authors:** Ye Qiu, Jianquan Zhang, Guangnan Liu, Xiaoning Zhong, Jingmin Deng, Zhiyi He, Bai Jing

**Affiliations:** 1 Department of Respiratory Medicine, the First Affiliated Hospital of Guangxi Medical University, Nanning, Guangxi 530021, China

**Keywords:** *Penicillium marneffei*, Trachea involvement, Tracheostenosis, Tracheal absence

## Abstract

**Background:**

*Penicillium marneffei* is the only dimorphic member of the genus and is an emerging pathogenic fungus that can cause fatal systemic mycosis. *Penicillium marneffei* disseminates hematogenously to other locations. *Penicillium marneffei* infection most commonly involves the skin, lungs, and reticuloendothelial system, including the bone, bone marrow, joints, lymph nodes, pericardium, liver, and spleen. Involvement of the mesenteric and central nervous systems has also been reported. Infection involving the trachea has not been previously reported.

**Case presentation:**

We herein report a previously healthy 28-year-old male farmer from Guangxi Province without HIV who became infected with *P. marneffei*. The infection primarily affected the trachea, resulting in structural damage to the cartilage, tracheal stenosis, and tracheal absence. The infection also involved the lungs and lymph nodes. After antifungal treatment and surgery, his symptoms, signs, and lung imaging findings showed significant improvement. This is the first such case report.

**Conclusion:**

*Penicillium marneffei* infection in normal hosts is characterized by an insidious onset, various clinical manifestations, and common misdiagnosis, leading to high mortality rates. *Penicillium marneffei* hematogenously disseminates throughout the whole body. This is the first reported case of *P. marneffei* infection involving the main trachea with subsequent structural damage to the tracheal cartilage, severe tracheostenosis, and tracheal absence.

## Background

*Penicillium marneffei* (PM) is a facultative intracellular pathogen that is capable of causing disseminated infection in both humans and wild bamboo rats. PM has been widely described in patients with HIV/AIDS as well as in other hosts, especially those with cellular immune defects [1]. Common clinical manifestations of PM infection are fever, weight loss, anemia, lymphadenopathy, hepatosplenomegaly, respiratory signs, and skin lesions [1,2]. PM infection most commonly involves the skin, lungs, and the reticuloendothelial system, including the bone, bone marrow, joints, lymph nodes, pericardium, liver, and spleen. Involvement of the mesenteric and central nervous systems has also been reported [1-3]. We herein report a case involving a 28-year-old man without HIV who developed a disseminated PM infection involving the main trachea. The infection resulted in structural damage to the tracheal cartilage, severe tracheostenosis, and up to 2 cm of tracheal absence. This is the first such case reported worldwide.

## Case presentation

**Figure 1:**
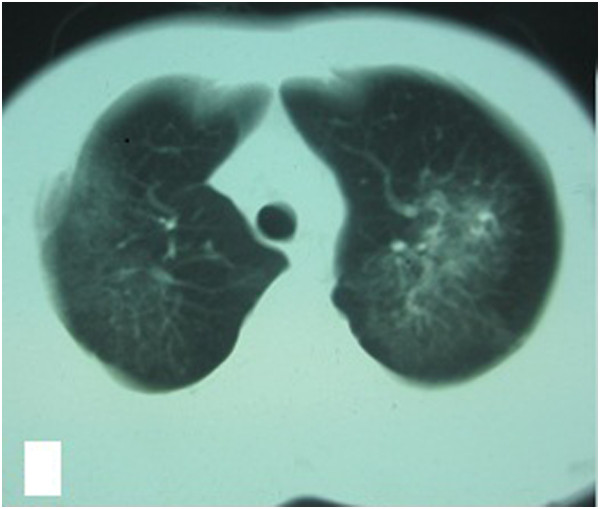
Chest HRCT showed plaques and exudation disseminated throughout both lung fields, especially the upper lung.

**Figure 2:**
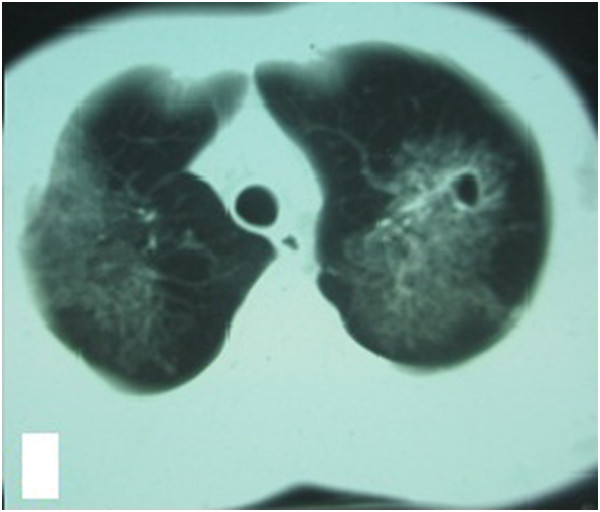
Chest HRCT showed that the lesions had grown in size and that a cavity was present in the left upper lung.

**Figure 3:**
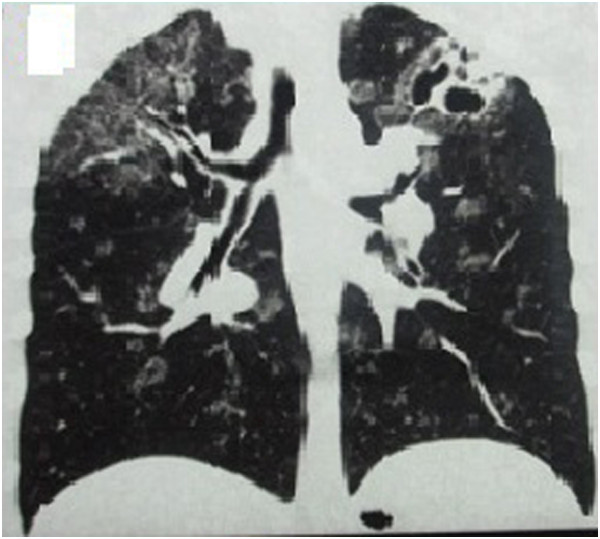
Chest HRCT showed that the lung lesions had worsened and that protrusions were present on the tracheal wall.

**Figure 4:**
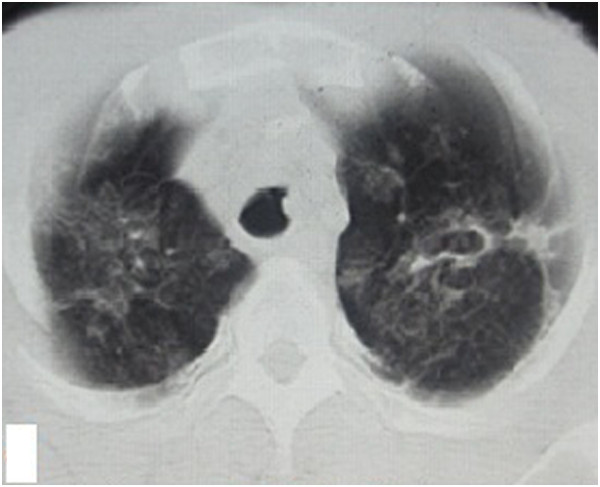
Chest HRCT showed that the lung lesions had worsened and that protrusions were present on the tracheal wall.

**Figure 5:**
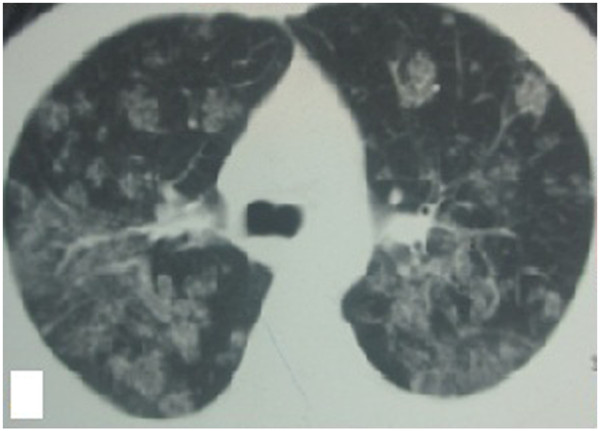
Chest HRCT showed that the lung lesions had worsened and that protrusions were present on the tracheal wall.

**Figure 6:**
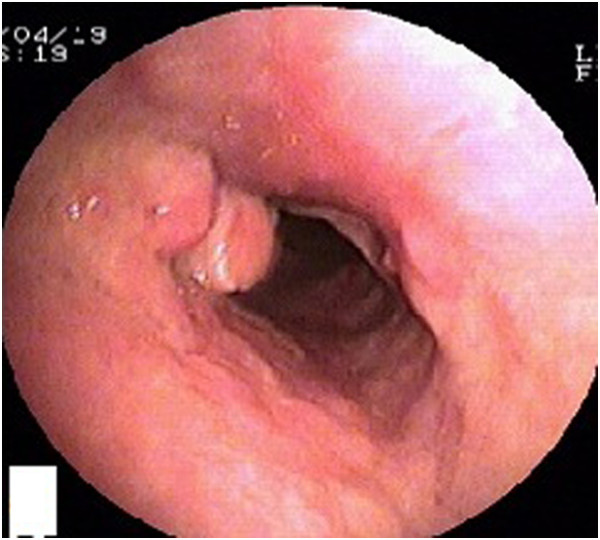
Bronchoscopy showed unevenness of the subglottic trachea and the presence of protrusions on the tracheal wall.

**Figure 7:**
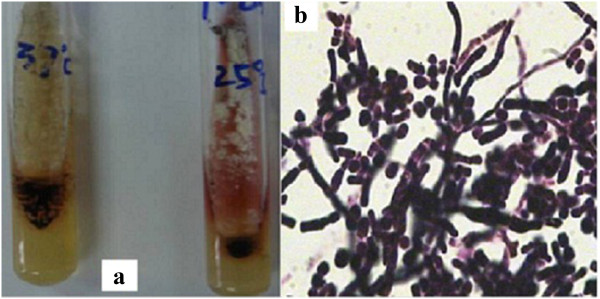
**The pathological examination results of the tracheal protrusions showed macrophages that were engorged with numerous yeast-like organisms 3 to 8 μm in diameter.** These yeast-like organisms were spherical to oval and had a transverse septum shown by the arrows **a** and **b** (period acid-Schiff staining, 400×).

**Figure 8:**
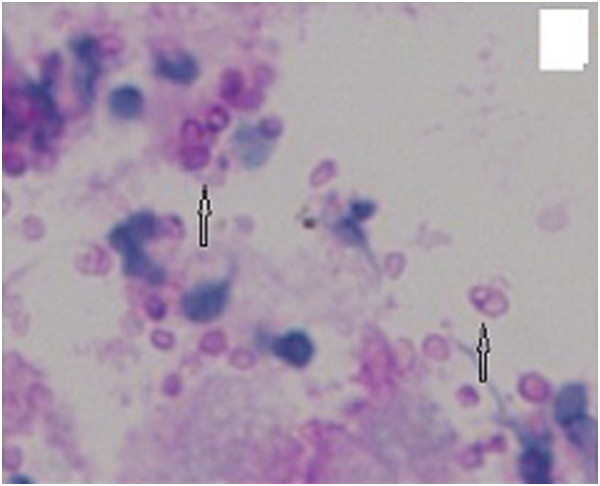
**(a) Bronchoalveolar lavage fluid and culture of the tracheal wall protrusions.***Penicillium marneffei* grows as a yeast at 37°C on Sabouraud dextrose agar but as a mold at 25°C on the same agar, exhibiting a soluble red pigment that diffuses into the medium. **(b)** The yeast form of *P. marneffei* has a characteristic morphology, including sausage-shaped yeast bodies shown by the arrow C.

**Figure 9:**
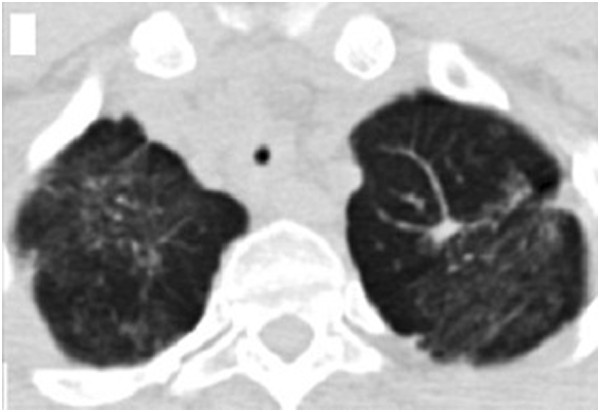
Chest HRCT showed a proximal tracheal occlusion, lower tracheal stenosis, and obvious absorption of the lesion.

A previously healthy 28-year-old male farmer from Guangxi Province, China was admitted to a local hospital because of blurred vision, numbness of the limbs, an intermittent fever, coughing, and sputum production in May 2012 after having killed several bamboo rats a few days previously. High-resolution computed tomography (HRCT) of the chest showed plaques, nodules, and exudation disseminated throughout both lung fields, especially the upper lung (Figure 1). A sputum smear, sputum culture, and cerebrospinal fluid culture were negative. Tuberculosis and neuromyelitis optica was confirmed as a clinical diagnosis according to the characteristic imaging findings and clinical manifestations. The tuberculosis was treated with isoniazid, rifampicin, pyrazinamide, and ethambutol, and the neuromyelitis optica was treated with prednisone (50 mg daily with gradual tapering until discontinuation). Three months later, his vision recovered and his numbness disappeared; however, his respiratory signs deteriorated. Post-treatment chest HRCT showed that the lesions were larger than their pretreatment size, and a cavity was found in the left upper lung field (Figure 2). A further 3 months later, the patient developed hoarseness accompanied by a sore throat. Laryngoscopy showed pharyngitis, but the patient did not respond to a 2-week course of antibiotics and his condition worsened. He was referred to our hospital on 16 April 2013. Laryngoscopy showed laryngeal and pharyngeal ulcers that were suggestive of laryngeal tuberculosis. Chest HRCT showed that the lung lesions had worsened and that protrusions on the upper tracheal wall had developed (Figures 3, 4 and 5). Bronchoscopy revealed unevenness of the trachea in the subglottic region and protrusions on the tracheal wall (Figure 6). Therefore, the antituberculosis treatment was continued with the addition of intratracheal instillation. Pathological examination of the protrusions on the tracheal wall showed atypical granulomas and microabscesses, but acid-fast staining results were negative. Periodic acid-Schiff staining revealed aggregates of macrophages that were engorged with numerous yeast-like organisms, 3 to 8 μm in diameter. These yeast-like organisms were spherical to oval and had a transverse septum (Figure 7). After 1 week, PM was isolated from a bronchoalveolar lavage fluid sample, and the tracheal protrusions were cultured (Figure 8a,b). The final diagnosis was disseminated PM infection involving the lungs, laryngeal tissue, mediastinal lymph nodes, and trachea. The antituberculosis treatment was changed to intravenous amphotericin B at an initial dose of 0.3 mg/kg daily with a gradual increase to 0.6 mg/kg daily as well as oral itraconazole at 200 mg twice daily. Two weeks later, the patient’s signs and symptoms were obviously improved; thus, only the itraconazole was continued, and the patient was discharged. Ten days later, he was referred to our emergency department because of severe dyspnea. Chest HRCT showed lower tracheal stenosis (Figure 9). Endotracheal intubation and tracheotomy were immediately performed. He was discharged after 1 week. Two months later, he was admitted again to our hospital because of the development of tracheostenosis.

**Figure 10:**
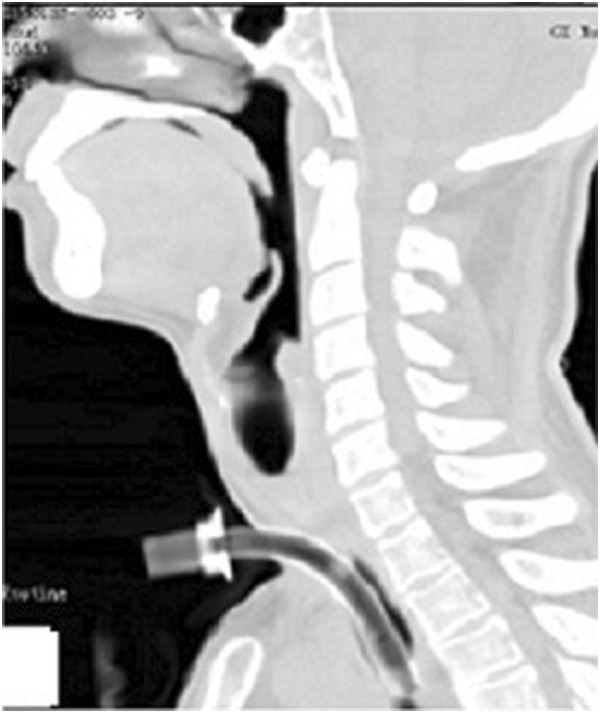
Lower segment tracheal collapse and tracheal absence of up to 2 cm between the tracheal occlusion and tracheal incision.

Physical examination revealed weight loss and a body temperature of 37.3°C. The chest, abdomen, superficial lymph nodes, and skin were normal. Chest HRCT showed a proximal tracheal occlusion, lower tracheal stenosis, and obvious absorption of the lesion compared with the previous HRCT (Figure 10). Laryngoscopy showed tracheal occlusion between the subglottic trachea and tracheal incision previously created for maintenance of respiration. Routine blood examination revealed a leukocyte count of 5.34 × 10^−9^/L, neutrophil percentage of 47.4%, and hemoglobin level of 116 g/L. His serum albumin level was 39.3 g/L. His CD4 T-cell count was 370/μL, C-reactive protein level was >200 mg/L, and erythrocyte sedimentation rate was 41 mm/h. His plasma beta-D-glucan measurement was high, but his galactomannan test results, aspartate aminotransferase level, alanine aminotransferase level, and creatinine level were all normal. He was negative for antistreptolysin O, antinuclear antibody, and rheumatoid factor. Blood and tissue microbiological cultures and HIV testing of blood samples were negative. These tests were repeated several times to ensure the reliability of the results.

**Figure 11:**
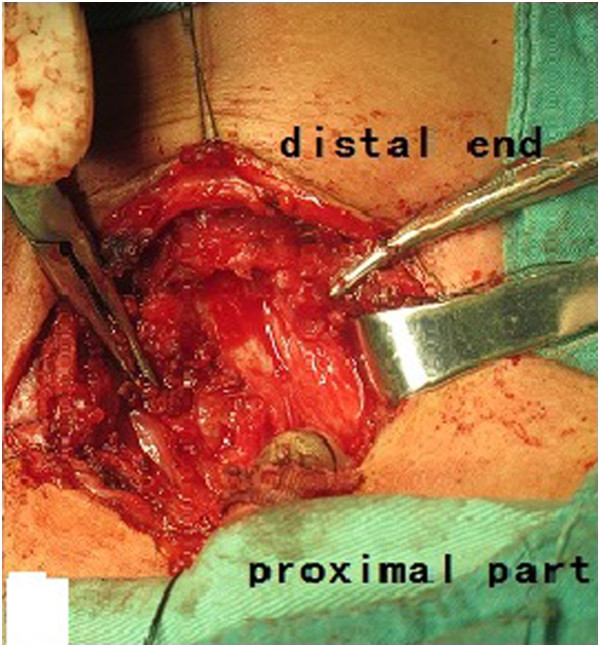
After treatment, the trachea was unobstructed and the lower tracheal stenosis and lesions showed significant improvement.

**Figure 12:**
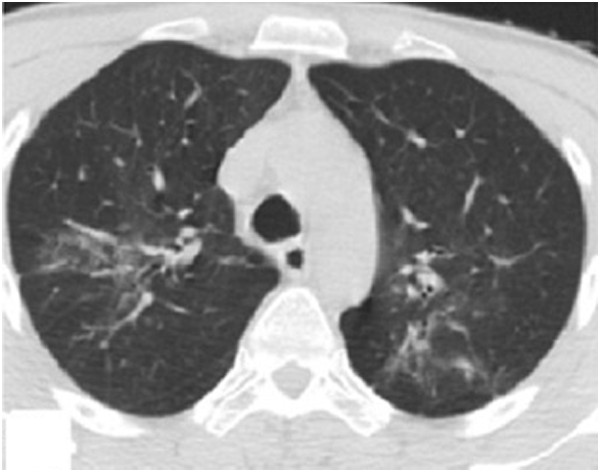
After treatment, the lower tracheal stenosis and lesions showed significant improvement.

**Figure 13:**
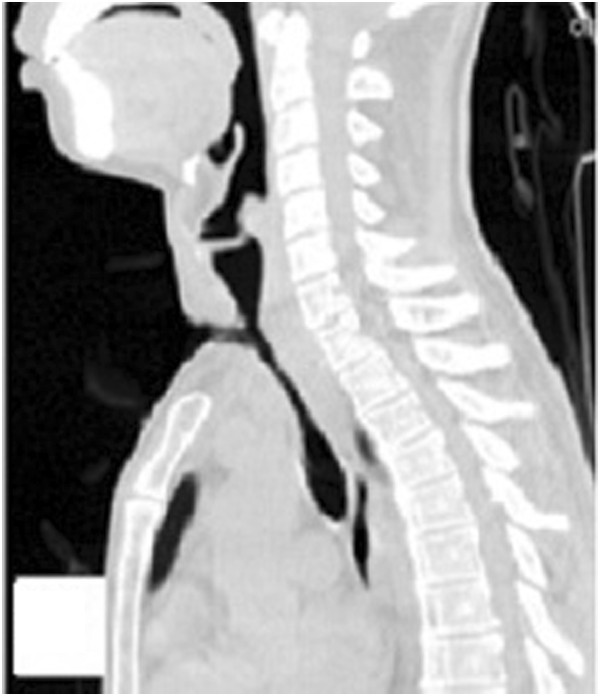
After treatment, the trachea was unobstructed.

Surgery was planned to treat the tracheal occlusion. During the surgery, we found tracheal collapse secondary to structural damage to the cartilage of the lower tracheal segment, as well as tracheal absence between the tracheal occlusion and tracheal incision (Figure 11). Specimens of the surrounding tissue were obtained for pathologic examination and culture. Tracheoplasty was converted to tracheal sleeve resection, tracheal cricoid anastomosis, and tracheostomy. The patient’s tracheal cannula was maintained to allow for breathing. The surrounding tissue cultures were negative. Pathological examination with periodic acid-Schiff, methenamine silver, and acid-fast staining was negative. The patient reported no postoperative dyspnea due to blocking of the tracheal stoma. The itraconazole was continued. The patient returned for follow-up 1 week postoperatively. His CD4 T-cell count had recovered to 745/μL, and his CD8 T-cell count was 412/μL. Chest HRCT showed that the trachea was unobstructed and that the tracheostenosis and lesions had significantly improved (Figures 12 and 13).

## Conclusions

PM disseminates hematogenously to other locations [1,2]. The reticuloendothelial system, skin, and lungs are the most commonly involved sites. PM can disseminate into the bone, bone marrow, joints, lymph nodes, pericardium, liver, spleen, mesenteric tissue, and even the central nervous system [1-3]. Healthy hosts infected with PM present with various symptoms. Patients with severe systemic inflammatory response manifests as fever, obvious increases in leukocyte and neutrophil counts, a high erythrocyte sedimentation rate, and a high C-reactive protein level. Moreover, weight loss, anemia, severe hypoproteinemia, and even multiple organ failure have been reported [4-6].

In this paper, we reported a case involving a healthy host without HIV or other underlying diseases who developed disseminated PM infection. Tests for antistreptolysin O, antinuclear antibody, rheumatoid factor, and HIV were negative. We found no possible causes of immunodeficiency. Although rare, PM has been reported in healthy hosts [4]. However, the present patient had risk factors for infection: he was living in an endemic area and had a history of contact with bamboo rats. After 5 months of antifungal treatment, his CD4 T-cell level recovered, indicating that he was not initially immunodeficient. PM grows as yeast bodies inside phagocytes. Immunosuppression after infection is a commonly encountered problem in affected patients. Causes of immune depletion may include prednisolone therapy, initial misdiagnosis, and disease progression.

The patient described herein exhibited symptoms different from those previously reported in the literature. His systemic inflammatory response was mild. The patient was misdiagnosed with tuberculosis as a clinical diagnosis because the lung imaging changes, symptoms, and protrusions on the tracheal wall were very similar to the characteristics of tuberculosis, which is a common disease in China. The long duration of misdiagnosis and mistreatment accelerated the progression of the disease, resulting in structural damage to the tracheal cartilage, tracheostenosis, and a tracheal absence of up to 2 cm. This is the first case of such developments in a patient with PM infection. Humans infected with PM can show three pathological patterns: suppuration, granuloma formation, or anergy with necrosis. The first and second patterns are more commonly seen in healthy hosts [7]. Because of the long duration of misdiagnosis of TB and prednisolone therapy in the present case, the PM infection involved the main trachea and subsequently resulted in severe tracheostenosis and tracheal absence. PM infection can result in the accumulation of massive numbers of neutrophils and the release of proteolytic enzymes in a local inflammatory response, causing tissue dissolution, suppuration, necrosis, and fibroplasia. These changes cause structural damage to the tracheal cartilage and cicatricial reparation, resulting in tracheal collapse, severe tracheostenosis, and tracheal absence.

PM infection is associated with high mortality because it is commonly misdiagnosed in the early stages [8,9]. PM is identified by culture, pathological examination, and cytological analysis [10]. PM grows as a yeast at 37°C, but as a mold at 25°C on Sabouraud dextrose agar, exhibiting a soluble red pigment that diffuses into the medium [1,8]. The yeast form of PM has a characteristic morphology, including a transverse septum and sausage-shaped organisms that can be demonstrated by pathological and cytological examination [8,10].

The differential diagnoses for PM include tuberculosis, histoplasmosis, lymphoma, cryptococcosis, panniculitis, molluscum contagiosum, and various other viral infections [8,10]. Tuberculosis is the most common misdiagnosis for the following reasons: (i) relevant literature and research on PM are limited; (ii) PM has been strongly emphasized as a common opportunistic infection of patients with AIDS; (iii) the signs, lung imaging findings, and pathological examination results are similar to those of tuberculosis; and (iv) in the early stages, there is a low concentration of viable lesions and a low culture-positive rate.

Penicilliosis is very susceptible to antifungal treatment. PM is susceptible to itraconazole and amphotericin B *in vitro*[11,12]. The current recommendation for patients with HIV infection is amphotericin B at a dosage of 0.6 mg/kg daily for 2 weeks, followed by oral itraconazole at a dosage of 200 mg twice daily for 10 weeks [11]. After 24 weeks of regular antifungal therapy and surgery, the symptoms, signs, and lung imaging findings of the present patient showed significant improvements.

PM infection in normal hosts is characterized by an insidious onset, various clinical manifestations, and common misdiagnosis, leading to high mortality rates. PM disseminates throughout the whole body via a hematogenous route. This PM infection involving the main trachea with subsequent structural damage to the tracheal cartilage, severe tracheostenosis, and tracheal absence is the first such reported case. The patient had a good response to antifungal treatment, but mild tracheostenosis remained after surgery. PM infection is characterized by a high rate of recurrence. Therefore, this patient will require a long follow-up duration to evaluate the long-term effects of the infection. There is no generally recognized treatment that is effective for PM infection in patients without HIV. The recommended treatment for healthy hosts requires further investigation.

## Consent

Written informed consent was obtained from the patient for publication of this case report and any accompanying images. A copy of the written consent is available for review by the Editor of this journal.

## Competing interests

The authors declare that they have no competing interests.

## Authors’ contributions

YQ made substantial contributions to the conception and design of the study; acquisition, analysis, and interpretation of the data; and drafting of the manuscript. JZ and GL made substantial contributions to the conception and design of the study; acquisition, analysis, and interpretation of the data; and critical revision of the manuscript for important intellectual content. JZ and GL also gave final approval of the version to be published and agree to be accountable for all aspects of the work in ensuring that questions related to the accuracy and integrity of any part of the work are appropriately investigated and resolved. XZ gave final approval of the version to be published. ZH participated in analysis and interpretation of the data and agrees to be accountable for all aspects of the work in ensuring that questions related to the accuracy or integrity of any part of the work are appropriately investigated and resolved. JD and JB conceived of the study, participated in its design, and helped to draft the manuscript. All authors read and approved the final manuscript.

## Authors’ information

Ye Qiu, Jianquan Zhang and Guangnan Liu considered co-first authors.

## Pre-publication history

The pre-publication history for this paper can be accessed here:

http://www.biomedcentral.com/1471-2334/14/242/prepub
